# Healthcare professionals’ perspectives on lung cancer screening in the UK: a qualitative study

**DOI:** 10.3399/bjgpopen20X101035

**Published:** 2020-06-10

**Authors:** Charikleia Margariti, Maria Kordowicz, Gillian Selman, Arjun Nair, Yvonne Akande, Azhar Saleem, Tiago Rua

**Affiliations:** 1 Research and Development Department, North East London NHS Foundation Trust, Essex, UK; 2 Lincoln International Business School, University of Lincoln, Lincolnshire, UK; 3 Guy’s and St Thomas’ NHS Foundation Trust, London, UK; 4 University College London Hospitals NHS Trust, London, UK; 5 Lambeth Clinical Commissioning Group, London, UK; 6 King’s Health Economics, King’s College London, London, UK; 7 Clinical Imaging and Medical Physics Directorate, Guy’s and St Thomas’ NHS Foundation Trust, London, UK

**Keywords:** lung cancer screening, lung neoplasms, primary health care, community care, secondary care, qualitative research

## Abstract

**Background:**

Lung cancer screening with low-dose computed tomography (LDCT) has been shown to decrease mortality. Low lung cancer survival rates in the UK, driven primarily by late-stage presentation, provide the impetus for implementing screening. Nascent guidance on screening in the UK recommends primary care case-finding. However, the potential impact and acceptability on primary care, and the opportunistic utilisation of other case-finding routes, such as pharmacies, smoking cessation services, and respiratory clinics, have not been fully explored.

**Aim:**

To explore healthcare professionals’ views and perspectives about lung cancer screening and their preparedness and willingness to be involved in its implementation.

**Design & setting:**

A qualitative study was carried out with semi-structured interviews conducted with GPs, pharmacists, staff from smoking cessation services within Southwark and Lambeth in London, and staff from respiratory clinics in Guys’ and St Thomas’ NHS Foundation Trust in London between April 2018 and December 2018.

**Method:**

Sixteen participants were interviewed and the interview transcripts were analysed thematically.

**Results:**

Participants described lung cancer screening as an important diagnostic tool for capturing lung cancer at an earlier stage and in increasing survivorship. However, the majority expressed a lack of awareness and understanding, uncertainty and concerns about the validity of screening, and the potential impact on their patients and workload.

**Conclusion:**

Study participants had mixed opinions about lung cancer screening and expressed their concerns about its implementation. Addressing these concerns by providing resources and effective and detailed guidelines for their use may lead to greater engagement and willingness to be involved in lung cancer screening.

## How this fits in

There is a gap in the literature about what healthcare practitioners in primary, secondary, and community care think and feel about lung cancer screening in the UK. GPs, pharmacists, and staff in smoking cessation services an respiratory clinics could provide a setting for the identification of eligible patients for lung cancer screening. This research provides data on healthcare practitioners’ perceptions, considerations, and attitudes towards lung cancer screening, and their suggestions in improving its implementation and outcome in the future.

## Introduction

Lung cancer is one of the four most common cancers for those aged >50 years.^1,2^ Survival rates in patients with lung cancer vary depending on the stage of lung cancer at the time of diagnosis. In the UK survival rates are among some of the lowest in Europe. Evidence suggests that this is owing to more patients being diagnosed at a late stage, where the chances of receiving curative treatment are substantially reduced.^3^ The CONCORD-2 study compared cancer survival from 67 countries between 1995 and 2009, this study showed that 5-year, age-standardised net survival for adults aged between 15 and 99 years in the UK was 9.6% compared with a range of 6%–20% across Europe.^4^

In approximately 90% of patients diagnosed with lung cancer, smoking is the main cause.^5^ Other risk factors for lung cancer include occupational or industrial exposure to carcinogens (including asbestos exposure), family history, previous history of malignancy, and previous radiotherapy treatment for some types of cancer.^6^

Even when lung cancer does cause symptoms there can be delays in the diagnosis, especially as lung cancer symptoms are non-specific and overlap with other respiratory illnesses, such as chronic obstructive pulmonary disease (COPD). Thirty-five per cent of cases are diagnosed after an emergency presentation, with 87% of those diagnosed at an advanced stage (stage III or IV),^6^ despite the fact that most people do present to their GP before a diagnosis of lung cancer is made.^7–9^ There is well-known evidence that the identification of lung cancer at a preclinical phase is associated with decreased lung cancer-related mortality.^10^ Currently, the standard chest radiograph (chest X-ray [CXR]), which is commonly used, is insufficiently sensitive or specific for the diagnosis of early stage lung cancer and reducing mortality.^11^ For this reason, interest in lung cancer screening with LDCT has accelerated over the last two decades.

The two largest randomised clinical trials of lung cancer screening with LDCT, the National Lung Screening Trial (NLST) in the US and the Dutch–Belgian NELSON study, have shown that LDCT could potentially reduce both lung cancer-specific and all-cause mortality.^12,13^ On the basis of the NLST results, the United States Preventative Services Task Force recommended lung cancer screening for eligible patients who are high risk at the end of 2013; despite this, uptake of screening has been derisory, at only about 3.9%. A key factor cited for the poor uptake is the reluctance of the clinical community to participate in shared decision making, underscoring the need for healthcare professionals to actively engage in, and be made a central part of, the process of lung cancer screening if it is to be implemented successfully.

In the UK, several trials^14,15^ have assessed the implementation of lung cancer screening, more recently using a model of lung health check (LHC) clinics to target high-risk individuals. Commonly used definitions for high risk are a risk of lung cancer of at least ≥1.51% over 6 years, and >2.5% over 5 years, based on validated multivariable risk prediction models.^16,17^ The imminent rollout of 10 national pilots as part of an NHS England-wide funded LHC programme is underpinned by a standard protocol that details the entire process, with case-finding performed through primary care.^18^ Given the late detection of lung cancer and low survival rates,^3,6^ a screening programme may have a maximum benefit in the UK.

Primary care will have a pivotal role to play in case identification for any national lung cancer screening programme in the UK, given the vital role it already plays in identifying patients with symptomatic lung cancer. Pharmacists too may play a key role in case identification, given their accessible nature within community care. Similarly, secondary care avenues can also provide an opportunistic patient identification point, particularly at respiratory and smoking cessation clinics. These two clinics would not only provide an opportunity for patients to discuss their concerns with a healthcare professional, but also address any potential barriers and negative attitudes they may have towards lung cancer screening.

As such, there is a clear and urgent need to seek and understand the views of the various healthcare professionals that could potentially contribute to lung cancer screening identification. To the authors' knowledge, this will be the first qualitative study in the UK examining the perspectives of healthcare professionals from primary, secondary, and community care about lung cancer screening programmes and their potential role within such a programme.

The aims of this study were:

- To examine the beliefs and attitudes of healthcare professionals within primary, secondary, and community care regarding lung cancer screening.
- To examine the perspectives of healthcare professionals within primary, secondary, and community care about their potential involvement in lung cancer screening.

## Method

A qualitative methodology, using in-depth interviews, was considered the most appropriate method for examining healthcare professionals’ perspectives and views about lung cancer screening in the UK. Interviews have been used previously to explore the perspectives of healthcare professionals and have been proven to be an effective data collection method.^19–21^

### Participants and recruitment

In order to capture their views and perspectives on lung cancer screening, participants were included if they were previously invited to participate in a High-Risk Lung Health Study as part of the Transforming Outcomes and Health Economics Through Imaging (TOHETI) programme at Guy’s and St Thomas’ NHS Foundation Trust and were any of the following:

- GPs in surgeries within Southwark and Lambeth Clinical Commissioning Group (CCG).
- Community pharmacists within Southwark and Lambeth CCG.
- Members of staff in smoking cessation clinics within Southwark and Lambeth CCG.
- Staff members at the respiratory clinic at Guy’s and St Thomas’ NHS Foundation Trust.

Participants were identified through the TOHETI research database as they had entered into a data-sharing agreement with the TOHETI lung cancer study at Guy’s and St Thomas’ NHS Foundation Trust.

Purposive sampling ensured a variation in age, healthcare discipline, and years of working experience and aimed to capture a wide range of views and perspectives on lung cancer screening. Participants were also recruited through professional contacts by using snowball sampling. This sampling strategy did not aim to recruit a representative sample of the population but to identify individuals who have lived specific experiences or have specific characteristics (that is, healthcare professionals),^22^ and to include participants outside of TOHETI’s lung screening study to minimise bias.

### Data collection

Interviews were conducted face to face or by telephone. Informed consent was obtained orally or in writing at the beginning of the interview, ensuring anonymity and confidentiality to participants. All the interviews were conducted at Guy’s and St Thomas’ NHS Foundation Trust premises and conducted by a single researcher.

The interview schedule has been developed based on previous literature and recommendations received by TOHETI’s research team. Table 1 shows the main topic guide.

**Table 1.** Topics for semi-structured interviews.

**Topic**	**Examples of questions**
Views about lung cancer screening	What do you know about the evidence around the benefits of lung screening?What is your opinion or views or thoughts about lung cancer screening?Do you have any considerations or concerns about lung health checks?
Views about being involved in lung cancer screening	Can you tell me if you have any concerns or reservations about being involved with lung cancer screening in the future?Do you believe lung cancer screening should be promoted and recommended by your service?
Suggestions or recommendations about lung cancer screening in the future	Do you have any suggestions or ideas about how to improve the engagement of your service in lung cancer screening?

The semi-structured interviews were guided by the topic guide (Table 1). This ensured that all the interviews had the same overall structure and that all the relevant issues were covered and discussed. No repeat interviews were conducted and no notes were recorded during the interview.

Previous evidence has shown that after 12 interviews the number of new emerging themes remained the same, suggesting that a sample of 10 to 12 interviews may be sufficient to enable the development of high-level, meaningful themes and useful interpretations.^23^ The point of data saturation was informed by a desire to ensure the richness and variety of the data and was determined to be 16 participants, once no new themes were emerging. However, the number of participants was also guided by the time and resource constraints of the study to enable its completion, given that the overall TOHETI programme was coming to an end.

Interviews were conducted between April 2018 and December 2018, and the average length of the interview was 12.3 minutes (range 12–20 minutes). The interviews were digitally audiorecorded and transcribed verbatim. Transcripts were checked for accuracy and anonymised, and were not returned to participants for further comments owing to participants’ busy schedule and time constraints.

### Analysis

The data were analysed thematically, a method that identifies, describes, and reports themes within the data.

For the thematic analysis, the guidelines developed by Braun and Clarke^24^ were applied. These guidelines suggest the following steps in conducting a thematic analysis: (a) familiarising one’s self with data; (b) generating initial codes; (c) searching for themes; (d) reviewing themes; (e) defining and naming themes; and, (f) producing the report.

The inductive process began with the line-by-line coding of each interview transcript. Codes were then combined and incorporated into broader themes employing software for the analysis of qualitative data (NVivo, version 11). Following the repeated reading of transcripts, key issues and themes emerging from the data were identified, shaping a draft thematic map by the interviewer and reviewed with the research team. Rigorous analysis was achieved by following a systematic procedure that permitted the transformation and organisation of the data into themes and enabled meaningful claims to be made.^21^

The research team acknowledged the potential researcher bias as the interviewee was employed by the TOHETI programme and precautions were taken, such as the use of a topic guide, to avoid question-order bias. Nonetheless, the schedule is framed from the perspective of utility towards a lung cancer screening programme and seeks answers to questions regarding considerations around future programme implementation, while assuming some prior knowledge to screening. Other means of assessing trustworthiness and credibility were the verbatim transcription of the interviews, the inclusion of quotes as representations, and illustrations for each theme.

The study was reported in accordance with the 32-item checklist of Consolidated Criteria for Reporting Qualitative Research (COREQ).^25^

## Results

In total, 16 healthcare professionals were interviewed (Table 2). Three key themes emerged from the analysis. Results are presented as these three overarching themes: awareness and understanding of lung cancer screening; optimism versus scepticism; and implications for guidelines, risk modelling, and organisational resources.

**Table 2.** Participant characteristics, *N* = 16.

Characteristics	**Frequency, *n***
**Sex**	
Male	10
Female	6
**Age, years**	
30–39	4
40–49	5
50–59	6
60–69	1
**Qualification**	
GP	10
Pharmacist	1
Staff in smoking cessation service (stop smoking specialist *n* = 1, coordinator *n* = 1)	2
Staff in the respiratory clinic (respiratory consultant *n* = 1, CNS *n* = 2)	3
**Years in current role**	
1–9	5
10–19	6
20–29	2
30–39	2
40–49	1

CNS = clinical nurse specialist.

### Awareness and understanding of lung cancer screening

All the participants described lung cancer screening as an important diagnostic tool for detecting lung cancer at an earlier stage. The provision of earlier diagnosis and treatment was also considered as important, as were improving clinical outcomes, survival rates, and care provision for the patients.

The majority of participants reported that they were aware of the concept of lung cancer screening from reading journals and online articles in their personal time; however, they did not feel confident about their knowledge and understanding about it. Participants were mostly aware that the majority of the research around lung cancer screening was conducted in the US and that currently, the NHS is not offering a national lung cancer screening programme in the UK. Only a few participants were informed about research programmes and pilot studies conducted in the UK, but they were not aware of the outcomes:

*‘My current understanding is that there is no formal lung cancer screening. Well the evidence of the big National Screening Cancer trial in the USA showed improved survival and mortality benefit. The evidence in Europe is a bit less strong and then in the UK, there isn’t anything that is specific, we still try to work out how to screen in terms of maximising benefit and minimising costs and match the national level screening.’* (Participant [P]14, doctor in the respiratory clinic)

### Lung cancer screening: optimism versus scepticism

Participants were ambivalent (positive or sceptical) about promoting lung cancer screening in the future. Although most practitioners considered lung cancer screening as beneficial in the NHS, they also expressed concerns about promoting it to their patients.

Several participants were positive about lung cancer screening and believed that it would be a beneficial tool to diagnose lung cancer at an early stage and to improve survival outcomes. They stated that screening should be offered when their patients meet certain criteria, such as age, smoking status, and family history, and to their patients at high risk. Some acknowledged that lung cancer screening will also benefit non-smokers, as there are other risk factors for lung cancer, such as passive smoking or exposure to environmental carcinogens:

*‘I think it will be a very useful tool and a very useful way to look at high-risk individuals, people who have been heavy smokers or might have other risk factors which predispose them to develop lung cancer.* […] *We’ve got screening pathways for many other conditions but we don’t have anything for lung cancer at the moment.’* (P8, GP)

Some participants stated that they were positive in promoting or identifying the patients for lung cancer screening in the future to detect disease in an early preclinical phase before symptoms develop. They believed that a screening tool for lung cancer could be a useful tool for healthcare professionals. In addition, they considered themselves capable of taking over and including it into their service.

Participants felt that although lung cancer screening could have a variety of positive outcomes, it could also have a negative impact on the patients and health services.

They considered it important to ensure that lung cancer screening will target, and be offered to the right target population. Participants noted that it should be offered to the high-risk population when appropriate, and healthcare professionals should avoid offering it to low-risk patients, causing them anxiety and stress:

*‘I guess it is crucially important to target certain groups. Obviously, with any screening, you have to have a cut-off between where you should stop to get people worried for no reason. If you are not really systematised, you are just going to encourage the relatively well and relatively low-risk smokers and therefore not identify that group that you are really going to get the most benefit from*.’ (P16, pharmacist)

The participants described concerns about the false positive incidents and false diagnoses that may cause unnecessary anxiety to the patients, including the detection of benign lung nodules. They stated that any future lung cancer screening should be tested and validated prior to its broader use to the general population:

*‘One* [concern] *is that there are quite a lot of false positives, mainly it feels like nodules, small lung nodules that are benign, that means that people get repeated scans and that can be quite an anxiety-provoking thing for the patients.’* (P3, GP)

*‘Well one issue with screening programmes is about raising anxiety and I think because benign lesions in the lung are not uncommon, I think this is a concern about finding benign lesions and then causing anxiety along the way.’* (P10, GP)

Also, some participants expressed their consideration about exposing patients to a high radiation burden and the frequency of recalling patients for follow-up screenings. They suggested that there should be guidelines and criteria about the eligibility of the patients and how often they should have a follow-up if needed:

*‘My biggest concern is the fact that you will do the screening, maybe you will do one round, two rounds, three rounds and then if you are picking up people in early to mid-fifties, what would you do then afterwards? Do we carry on screening them forever? Or we do it again in ten years’ time?’* (P15, staff in smoking cessation service)

Participants noted that in some instances the negative outcome of the screening may be misleading and provide a false relief to the smokers who will not quit smoking:

*‘* *I think another slight concern is how the message of the negative screenings would be interpreted. Will patients think “that’s fine, I don’t have cancer” and continue smoking? We have to think about this issue.’* (P5, clinical nurse specialist)

In addition, some GPs expressed their concerns and hesitance about being responsible for lung cancer screening in their service. Without having the appropriate and sufficient resources, taking over this new type of screening could potentially increase their workload:

*‘I think as a GP, given that the workload for us is huge as it is now, a lung cancer screening might add on more work pressure because I wonder if the population that might need to be screened will be quite large, so it will be time pressure for the GP to be doing this themselves*.*’* (P12, GP)

Others stated that GPs will need extra training and education about this screening and should be well-prepared to deal with a patient’s anxiety:

*‘As with all screening involves education on the part of the GP, to encourage patients to attend screening but also have enough knowledge to know and advise the patient what the screening involves and that obviously takes some time, we need to be able to deal with the increased anxiety and deal with the anxious patients so it’s not only about time and resources.* *’* (P1, GP)

### Implications for guidelines, risk modelling, and organisational resources

Most healthcare professionals agreed that clear and detailed guidelines about this type of screening should be provided to them in order to deliver it successfully in their service. It will be essential for its successful implementation to provide guidelines that all healthcare professionals could understand and follow:

*’* *I think the lung cancer screening pathway has to be clear and easy for the whole primary care team to understand its guidelines, it’s not only for the GPs but also frontline staff like primary care navigators and healthcare assistants who also have a role in promoting screening programmes. So the pathway has to be clear and easy to understand for the patients as well.* *’* (P9, GP)

They also suggested that there should be scientific evidence about implementing a risk model of eligibility. Participants described the importance of eligibility criteria that will not only focus on the patient’s smoking habits but will also acknowledge environmental factors and family history, and identify patients who are at the greatest risk for lung cancer incidence, and the potential morbidity and mortality that goes with it. In addition, they highlighted the importance of offering screening to vulnerable populations:

*‘A lot of our patients, a lot of people with lung cancer, it’s lower socioeconomic status, we get a lot of problems with the homeless population,* […] *and people who are quite hard to encourage to come to clinic in any case let alone to have an extra appointment for lung cancer screening or just take the initiation and come themselves. So we have to make sure we can encourage this hard-to-reach population for screening.* *‘* (P5, clinical nurse specialist)

## Discussion

### Summary

Healthcare professionals considered lung cancer screening as an important and useful diagnostic tool in order to diagnose lung cancer at an early stage. Most of their knowledge was derived from research studies and evidence in the US. Participants had ambivalent views about the use of lung cancer screening in their service.

On the one hand, many weighed up the benefits of this type of screening, they were positive about it and acknowledged its beneficial impact in improving survivorship rates for patients with lung cancer. On the other hand, they felt cautious and uncertain about the issues that may arise owing to its incorrect implementation or overuse, such as radiation, false positive results, and patients’ anxiety. In addition, they were concerned over whether they had the required resource to identify patients for screening, in light of an ever-increasing workload. However, the participating healthcare professionals suggested that with the right guidelines, risk modelling, scientific evidence, and financial resources they would be more confident to provide this screening to their patients.

### Strengths and limitations

The main strengths of this study are that it provides information about the perspectives of lung cancer screening in the UK. Adopting a qualitative approach allowed a detailed exploration of the attitudes and feelings of the healthcare professionals on this topic. The use of semi-structured interviews for data collection enabled participants to share personal experiences that might not have emerged through questionnaires. A sample of participants from different healthcare settings facilitated a range of responses in the recruited group and was a novel aspect of the current study.

Despite these efforts, sampling strategies such as snowballing can result in viewpoints that are not entirely diverse. Healthcare professionals were sampled from across one geographical area, using homogeneous purposive sampling by seeking only healthcare professionals who have been invited to participate in TOHETI’s lung project, rather than by random sampling. Arguably, recruiting from a sample of GPs and pharmacists who were already involved with TOHETI’s lung screening study may have created a bias towards those already open to the idea of lung cancer screening. Snowball sampling through the use of contacts widened the participant pool beyond TOHETI. Further, participants also expressed negative views towards screening and these are presented here. In addition, the majority of the participants were GPs; however, the aim of this study was to capture overall perspectives and not to examine the differences and similarities between each clinical group.

It is likely that healthcare professionals who were interested in the topic of lung cancer screening in particular volunteered. This may mean that their views were somewhat more informed on this topic than others who did not participate. In addition, the awareness around lung cancer screening may reflect the perspectives of the local NHS services and it could vary across the UK where primary care has been actively engaged in screening programmes, affecting the generalisability of the findings.

### Comparison with existing literature

To the authors' knowledge, there is no existing qualitative research conducted in the UK that addresses the perspectives and preparedness of healthcare professionals’ towards lung cancer screening across primary, secondary, and community care. The majority of previous studies examining the perspectives of lung cancer screening were carried out in the US, using quantitative methods, and concerned with the views of patients, primary care physicians, and specialists. However, the findings of the current study are consistent with a handful of prior qualitative studies of lung cancer screening.

Consistent with the views of primary care providers in a US study,^26^ healthcare professionals in this study were generally positive about a potential lung cancer screening programme, but had mixed views about their preparedness. A previous qualitative study in the US showed that GPs suggested that training and ongoing support were fundamental in order to accommodate a new diagnostic tool for lung cancer in their daily practice.^27^ Similar views were expressed by GPs in this study, suggesting primary healthcare professionals may need extra resources and training prior to integrating lung cancer screening in their daily routine. Previous findings also suggested that primary care clinicians need further education about lung cancer screening in order to determine when this screening is appropriate or not.^28^

Financial support for lung cancer screening was an important factor for healthcare professionals in this study in order to take over this new screening in their service. Similarly, in a US study, financial reimbursement was an important factor that influenced primary care physicians’ decisions about screening patients for lung cancer.^28^

In line with O’Brien *et al* *’s* ^26^ study, participants also suggested that scientific evidence about potential benefits and risks of the screening, along with detailed guidelines, should be available to them. In addition, O’Brien *et al* ^26^ highlighted the need to educate patients about the purposes of lung cancer screening in order to participate in shared decision making.

The need for specific and clear lung cancer screening eligibility criteria was consistent with the findings of a previous US study,^29^ where primary care providers were unsure about the screening eligibility criteria and guidelines.

It should be noted that all the above findings from the US studies are based on healthcare professionals’ experiences and perspectives about an existing lung cancer screening programme in the US, whereas in this study healthcare professionals expressed their views and concerns regarding a potential lung cancer screening programme that does not currently exist in the UK.

### Implications for research and practice

The findings of this study contribute to the understanding of how healthcare professionals in the UK can be supported to provide and be engaged with lung cancer screening. Although some of the participants acknowledged the health benefits of screening, the need to address the uncertain knowledge about cancer screening, and to clarify the role of specific healthcare services, is apparent.

Concerns about the negative consequences of referring a patient for lung cancer screening in some instances suggests that healthcare professionals need detailed and scientific evidence-based guidelines, and training about its appropriate use. The majority of suggestions mentioned by the participants have been previously addressed and successfully incorporated into screening programmes for other types of cancer (that is, breast cancer), such as scientific evidence for eligibility criteria, risk modelling, and minimising overdiagnosis.^30^

Participants particularly highlighted the need for specific inclusion criteria for identifying the right population, and to avoid its misuse or overuse impacting negatively on patients. It may be necessary to provide financial incentives and workforce support to general practices, pharmacies, smoking cessation services, and respiratory clinics in order to ensure that they have the sufficient resources to implement and provide lung cancer screening. Finally, it is clear from this study that, presently, healthcare professionals across different healthcare services remain ambivalent about the implementation of lung cancer screening.
